# Examining Increased Ritalin and Adderall Use Among Low-Income People Who Use Drugs: Mixed Methods Study

**DOI:** 10.2196/99947

**Published:** 2026-09-01

**Authors:** Ian David Aronson, Robert Quiles, Anthony Cramer, Chunki Fong, Alex S Bennett

**Affiliations:** 1 Center for Technology-based Education and Community Health (TECH), NDRI-USA, 31 W 34th St, New York, NY, 10001, United States, 1 (212) 845-4444; 2 School of Global Public Health, New York University, New York, NY, United States

**Keywords:** Ritalin, Adderall, opioids, fentanyl, stimulants, adulterants, artificial intelligence, AI

## Abstract

**Background:**

Popular discourse often frames prescription stimulants Ritalin and Adderall as drugs for teens and emerging adults with greater financial resources (eg, students or young professionals), while illicit stimulants such as crack or methamphetamine are often considered drugs for older adults with lower incomes. Crack and methamphetamine are also frequently used in combination with opioids to offset the sedating effects of adulterants such as fentanyl and xylazine in the unregulated drug supply. This combination of stimulant and opioid use creates additional overdose risks and may require new public health approaches. Our team posited that creating effective messages to protect against overdose in the context of stimulant use entailed first developing a better understanding of which stimulants people are using in combination with opioids.

**Objective:**

While conducting formative research to develop a new intervention, our team sought to examine prescription stimulant use among participants, including middle-aged, lower-income, and homeless people. This entailed first ascertaining the prevalence of Ritalin and Adderall use among participants, and then asking people why they used them. We also sought to implement a novel AI-assisted coding methodology and to write up the steps we took as a replicable model that other research teams could readily use to facilitate their own data analysis.

**Methods:**

We collected substance use screenings during 3 waves of data collection in 2025 (N=102 participants). In early 2026, we conducted 16 qualitative interviews with people who reported using Ritalin or Adderall. We then conducted mixed methods analyses to examine reported substance use, including the use of opioids and Ritalin or Adderall in combination, as well as potential relationships between using opioids with prescription stimulants and reporting a desire to stop using drugs. After conducting the interviews, we implemented a new hybrid methodology using AI tools to code interview transcripts and generate detailed reports accompanied by supporting quotes.

**Results:**

Participants described using Ritalin or Adderall to manage negative effects of increasingly powerful opioids, including oversedation and withdrawal symptoms. A subset of participants who reported using both Ritalin or Adderall and opioids were 3.5 times more likely to agree or strongly agree with the statement “I want to stop using drugs but I need help to do that” compared to those who did not report using these substances in combination (19/22, 86.4% vs 18/28, 64.3%; odds ratio 3.52, 95% CI 0.83-14.89; *P*=.09). Although not statistically significant, this difference may prove practically significant and merits further study.

**Conclusions:**

The use of prescription stimulants appears to be increasingly common among new populations of people who use opioids, including those who report very low incomes and even homelessness. Additional research is warranted to examine how this type of polysubstance use may require different types of prevention strategies.

## Introduction

In New York and nationwide, people are increasingly using stimulants to counteract the effects of more potent opioids [1], which has led to a “fourth wave” of the overdose crisis [2]. In New York State, overdose deaths involving fentanyl and psychostimulants such as methamphetamine increased approximately 5.6 times between 2018 and 2022 (from 89 to 500), while overdose deaths involving fentanyl and cocaine nearly tripled (from 786 to more than 2200) in the same period [3]. Nationally, from 2018 to 2023, the largest increase in overdose deaths was similarly among people who used both stimulants and opioids [1].

Although there have been modest decreases in overdose deaths nationwide and in New York, where our research is conducted, overdose remains particularly deadly in lower-income neighborhoods and among homeless people. In New York City, overdose deaths decreased in neighborhoods with high poverty rates much more slowly than in wealthier areas [4]. Homeless people face especially high overdose risks due to “chaotic, dangerous” circumstances [5] and because they often lack the privacy to engage in safer drug use practices such as using smaller initial test shots (“try before you fly”) or drug-checking strips to test for fentanyl and xylazine.

Research by our team conducted across the city with a sample of largely low-income people, many of whom reported earning less than US $10,000 per year, is generally consistent with these observed trends. We found increased use of opioids and stimulants in combination among a sample of people who use drugs in New York City. Formative research by our team for a project designed to increase protection against overdose found that, while some participants reported using stimulants but not opioids, all the participants in our study who reported using opioids also reported concurrent stimulant use [6]. However, our findings differ from those of prior research in that, while other studies have focused on the use of opioids in combination with cocaine (in both powder form and as crack) and methamphetamines (eg, [1-3]), our recent findings show an increase in the use of opioids with the prescription stimulants Ritalin and Adderall.

Methylphenidate (Ritalin), which entered clinical practice in the 1950s, and Adderall, a mixture of amphetamine salts approved in the United States in 1996, rapidly became two of the most frequently prescribed medications for attention-deficit/hyperactivity disorder (ADHD) [7,8]. Diversion of prescription stimulants for nonmedical use has been documented for decades [9,10]. However, our preliminary findings stand out because they appear to show differences not only in *what type* of stimulants people use with opioids (prescription Ritalin or Adderall vs unregulated methamphetamine and powder cocaine or crack) but also the *demographics* of who uses prescription stimulants. Prior research generally describes people who use Ritalin or Adderall as White [11,12], wealthy [10,13], and younger [14,15] individuals. However, the sample we recruited self-identified as older, largely African American and Latino individuals and included lower-income and unhoused participants, demographics not generally associated with prescription stimulant use in the literature.

Thus, in light of apparent differences between our initial findings and prior research, the primary aim of this study was to conduct a mixed methods examination of prescription stimulant use among participants recruited for our formative work. In particular, we sought to examine how participants’ Ritalin or Adderall use might align with existing research (eg, whether people were, in fact, using these stimulants to mitigate the effects of more potent opioids) and how it might differ (ie, whether the demographics of prescription stimulant use actually appeared to be shifting). Furthermore, we sought to examine whether there might be any relationship between reporting use of Ritalin or Adderall in combination with opioids and a reported desire to stop using drugs.

As a secondary aim, we sought to implement a new AI-assisted methodology to help our team code and analyze interview transcripts and to write up the steps of this new method so that it can be implemented by other research teams who might be interested in incorporating AI tools into their own data analysis. We had been experimenting with AI tools to create intervention content and hypothesized that, if properly supervised by experienced, thoughtful members of our team, AI could be used to facilitate interview coding and the extraction of relevant quotes.

Numerous studies have examined the use of AI tools to code and analyze qualitative data (eg, [16]), including studies that carefully compared AI-coded interviews to human-coded ones [17,18]. This prior research has established that AI can effectively complement human effort in different phases of coding [17] and analysis [16]. It has also established that augmenting traditional qualitative analysis with AI, for example, using AI as an additional coder or to reduce potential human bias, offers a promising strategy so long as humans provide necessary oversight [18]. However, our search of peer-reviewed articles did not show that other researchers had used commonly available conversational AI models to implement the type of iterative, closely supervised methodology we developed for the current study (our full team created and revised an interview guide, then multiple team members collaboratively walked an AI through the coding of transcripts and extraction of quotes, reviewing and critiquing the AI’s work at each step). Therefore, the intent of the current study was never to compare human coding with AI coding but, instead, to present a model of AI support for human coding that other researchers can adapt to their own work across the behavioral sciences. Accordingly, we explain the details of our method in the following sections and include our prompts in Multimedia Appendix 1.

## Methods

### Preliminary Recruitment and Substance Use Screening

In 2025, we used venue-based sampling to collect 3 waves of data. From February to December, interviewers who had extensive experience working with people who use drugs recruited participants aged 18 years or older in parks and other public areas around New York City. Eligibility criteria included reporting illicit substance use in the previous 30 days. During regularly scheduled data collection shifts (eg, 9 AM to 5 PM on Wednesdays, Thursdays, and Fridays), interviewers traveled to different parts of the city. Our protocol specified recruiting new participants for each wave. On occasions when interviewers returned to a location where they had previously recruited participants, they explained to people they recognized from prior encounters that anyone who had already participated could not do so more than once.

Interviewers reported that they approached approximately 20 individuals who declined to participate for various reasons, including lack of time and interest. Approximately 50 additional people were approached but were not eligible to participate because they reported not using illicit substances.

All participants used tablet computers to complete a substance use screening based on the National Institute on Drug Abuse Modified Alcohol, Smoking, and Substance Involvement Screening Test (ASSIST) [19,20] that asked participants, among other questions, how many days out of the previous 30 they had used “Stimulants (eg, Ritalin / Adderall)” without a doctor’s prescription or in greater amounts or longer than prescribed. The screening contained separate questions for cocaine or crack and “methamphetamine (like speed).”

Wave 1 interviews explored barriers to engagement with safer drug use services and practices (ie, not using drugs alone). Wave 2 examined participant response to different configurations of potential intervention content (eg, photorealistic vs cartoon images and sample messages). Participants in wave 3 responded to a set of pilot measures examining attitudes toward safer drug use. One of the wave 3 items asked participants to respond to the statement “I want to stop using drugs but need help to do that” by clicking on a 5-point Likert-type scale with options labeled “strongly disagree,” “disagree,” “neither agree nor disagree,” “agree,” and “strongly agree.” All pilot measures were administered via tablet computer.

In 2026, our team recruited a new set of participants, wave 4, to examine the use of prescription stimulants. Participants in wave 4 were eligible for inclusion if they reported using Ritalin or Adderall without a doctor’s prescription or in greater amounts or longer than prescribed at least once in the previous 30 days. Interview questions focused on details of their Ritalin or Adderall use.

Study team members (interviewers and investigators) possessed a wide range of lived experiences of substance use and harm reduction engagement. A chief objective of our team was to ensure that all procedures were carried out in a dignified setting free of judgment and stigma.

### Ethical Considerations

The research protocol, including interview questions, eligibility criteria, and recruitment and interview procedures, was approved by the Biomedical Research Alliance of New York, which serves as the single institutional review board for this study (ID 24-354-1720). Participants provided verbal informed consent and were paid US $20 in cash.

### Transcript Preparation

The 2025 interviews were professionally transcribed by a company that offered “human-generated transcription services.” Research staff reviewed each transcript line by line while listening to the original audio recordings.

Transcripts for the 2026 interviews were automatically generated from the recorded audio files using Adobe Premiere software. These automated transcripts were similarly checked for accuracy by the 2 staff members who had conducted the interviews and by the study principal investigator (PI), who listened to the original audio files while reading along line by line. Any transcription errors were immediately corrected. During weekly meetings, our team discussed transcript quality and preliminary interview findings. This process helped us develop a shared familiarity with our data. After all the transcripts had been reviewed, the full research team drafted and revised a list of qualitative codes.

### AI-Assisted Coding

The full research team collaboratively developed a codebook based on study objectives and the interview guide for the wave 4 interviews. The PI uploaded this guide to Claude Sonnet 4.6 (Anthropic), along with a set of prompts instructing Claude to code our transcripts and then generate a new Microsoft Word document for each transcript detailing how the codes had been applied. Next, the PI uploaded a sample transcript for Claude to work with and reviewed the resulting Microsoft Word document that Claude created. The PI provided multiple rounds of feedback to Claude in the form of additional prompts to refine the coding process (eg, explaining the difference between parent and child codes and noting that some codes were applicable only to current Ritalin or Adderall use, whereas others were intended solely for discussions of initial use). After each round of new prompts, the PI asked Claude to code the transcript again. Once it appeared Claude was coding the transcript as desired, the PI repeated the process with a second transcript to confirm that this was the case. The prompts are included in Multimedia Appendices 1 and 2.

The PI then uploaded the remaining transcripts and asked Claude to code them one by one and again output a separate document for each transcript describing which codes had been applied to each section of the interview. Next, the PI asked Claude to create a report identifying themes that emerged among the full collection of coded transcripts and provide interview quotes illustrating each theme.

### Full-Team Review

Once all the documents were completed, the PI shared the coded transcripts with the 2 team members who had conducted the interviews. He asked them to evaluate the coded transcripts line by line to verify that Claude had coded the interviews as we intended and add or remove codes as appropriate. The interviewers did not find any data that had been coded incorrectly but did have some suggested revisions, for example, asking Claude to include not only selected participant responses but also interview questions if they might provide important context. Additionally, the interviewers noted that some quotes that Claude had selected contained dialogue between the interviewer and participant but did not indicate which words were spoken by whom. Our team agreed that these issues could be resolved by providing Claude with additional prompts.

The full team then reviewed the report that Claude generated after coding the entire set of transcripts. There was consensus that the report was very thorough, included accurately coded and categorized excerpts, and did not contain anything problematic (eg, irrelevant quotes or hallucinated data). Staff also manually compared the report to the full set of transcripts to determine whether any relevant material had been left out. They identified some relevant quotes that were applicable to our research but had not been included in the report (although the PI noted that other excerpts already included in the report addressed similar themes). Staff also noticed that the AI at times used very short versions of quotes and left out potentially relevant text. We discussed whether Claude should be prompted to “exhaustively” include all relevant quotes or process the data more selectively and only include a limited number of shorter excerpts (we collectively decided on a more selective approach and strongly agreed on the value of a hybrid methodology in which team members carefully compared AI-selected excerpts to the full transcripts for quality assurance).

As an additional validity check, the 2 interviewers manually coded a subsample of the interview transcripts. Using MAXQDA qualitative data analysis software (VERBI GmbH), the interviewers coded a total of 4 transcripts. Their coding aligned strongly with that of Claude. No significant discrepancies were identified. As described above, there were instances when Claude excerpted a shorter section of a quote while the interviewers believed that a longer version would contain additional helpful details.

Prior research has shown that AI can be used effectively to assist trained researchers in each phase of qualitative content analysis [16]. Studies also show that AI tools can substantially reduce coding time without sacrificing quality [21], especially when applying codes from a human-generated codebook [22]. Our work on this study, which entailed using AI to apply codes from a codebook that had been carefully developed by our full research team, appears to support these earlier findings.

## Results

### Descriptive Data

#### Waves 1 to 3

In 2025, we collected 3 waves of data and screened a total of 102 eligible participants (an additional 39 participants completed the automated substance use screening and were identified as ineligible either due to a lack of illicit substance use or because they reported an age younger than 18 years). Among the 102 eligible participants, the mean age was 45 (SD 14.1) years; 80 (78.4%) identified as male, 19 (18.6%) identified as female, and data for 3 (2.9%) were unknown or not reported. Most (65/102, 63.7%) had completed high school or secondary school, and most (70/102, 68.5%) reported an annual income of less than US $10,000. The percentage of participants reporting Ritalin or Adderall use increased during each wave of recruitment, although the difference was not statistically significant. Table 1 provides more details.

**Table 1.** Ritalin or Adderall use by data collection wave.^a^

	Number of participants per wave	Participants reporting use of Ritalin or Adderall n (%)
Wave 1	30	10 (33.3)
Wave 2	22	9 (40.9)
Wave 3	50	24 (48.0)

a Overall significance: *P*=.43.

#### Wave 4

In March 2026, we recruited 16 new participants who reported using Ritalin or Adderall in the previous 30 days other than as prescribed by a physician. The mean age was approximately 40 (SD 12.1) years; of these 16 participants, 14 (87.5%) were male, and 2 (12.5%) were female. Most (13/16, 81.3%) had completed high school or secondary school, and more than half (8/13, 61.5%) reported an annual income of less than US $10,000.

There were no statistically significant demographic differences between participants recruited in 2025 (waves 1 to 3) and those recruited in 2026 (wave 4).

### Quantitative Analyses

Among participants in the first 3 waves (n=102), those who reported Ritalin or Adderall use were significantly more likely to also report opioid use within the previous 30 days (35/43, 81.4%) compared to participants who did not report Ritalin or Adderall use (32/59, 54.2%; odds ratio 3.69, 95% CI 1.47-9.29; *P*=.006; Table 2).

Participants in wave 3 who reported using both Ritalin or Adderall and opioids within the previous 30 days were 3.5 times more likely to agree or strongly agree with the statement “I want to stop using drugs but I need help to do that” compared to those who did not report using these substances in combination (19/22, 86.4% vs 18/28, 64.3%; odds ratio 3.52, 95% CI 0.83-14.89; *P*=.09; Table 3).

**Table 2.** Ritalin or Adderall and opioid use among participants in waves 1 to 3 (n=102).^a^

Overall, n (%)	Opioid use reported, n (%)	No opioid use reported, n (%)
Ritalin or Adderall Use Reported	43 (42.2)	35 (81.4)	8 (18.6)
No Ritalin or Adderall Use Reported	59 (57.8)	32 (54.2)	27 (45.8)

a Odds ratio 3.69, 95% CI 1.47‐9.29; *P*=.006.

**Table 3.** Participant agreement with “I want to stop using drugs but I need help to do that” by type of substance use (wave 3 only; n=50).^a^

	Overall, n (%)	Agreed or strongly agreed, n (%)	Did not agree or strongly agree, n (%)
Ritalin or Adderall and opioid use reported in combination	22 (44)	19 (86.4)	3 (13.6)
No reported use of Ritalin or Adderall and opioids in combination	28 (56)	18 (64.3)	10 (35.7)

a Odds ratio 3.52, 95% CI 0.83‐14.89; *P*=.09.

### Interview Findings

#### Using Prescription Stimulants to Manage Opioid Effects, Including Oversedation and Withdrawal

While Ritalin and Adderall are generally prescribed to treat ADHD and sometimes for weight loss or to treat narcolepsy [14], participants consistently reported using prescription stimulants to manage the negative effects of unregulated opioids. This included balancing or evening out the potentially deadly oversedation effects of fentanyl, xylazine, and other adulterants that are overwhelmingly present in today’s opioid supply. In the words of a White Hispanic male participant aged 49 years:

...the fentanyl is too strong so I use an Adderall or another thing to wake up.

Other participants made similar comments and described using Adderall when they needed to complete a task or show up for an appointment.

Similarly, many participants described using Adderall to prevent them from becoming completely sedated or spending extended periods in a fully sedated state:

I’m [on] a high dose on methadone, and um, sometimes I do heroin, so I need something to come up. I don't want to be down all day like that.

[Black Hispanic male participant; aged 57 years]

This type of adaptive and functional use appears to have become increasingly common as opioids have become more potent in recent years due to potentially deadly adulterants. Participants also reported using prescription stimulants to decrease the amount of opioids they used (which they specifically named as fentanyl and “tranq” for xylazine or described more generally as “down”) while also preventing or lessening withdrawal symptoms:

It makes you do a little less. You know what I’m saying? Fentanyl. Or tranq.

[American Indian or Alaska Native male participant who did not report ethnicity; aged 42 years]

Because it avoids me feeling sick with the down till I get the down. You know, if I can't have the down...I feel okay, without having to do opiates, five hours a day, you know, I could go ten hours or 12 and even half a day without even doing opiates.

[White Hispanic male participant; aged 39 years]

The above quote appears to indicate not only that people are using stimulants to balance out effects such as oversedation, which has been established in prior studies (eg, [1]), but also in an attempt to control withdrawal symptoms. Adulterated opioids can precipitate multiple types of withdrawal if people stop using them. In addition to withdrawal from the opioid itself (ie, heroin), adulterants such as fentanyl, benzodiazepines, and medetomidine can each cause their own painful, sometimes life-threatening withdrawal symptoms. Moreover, the effects of fentanyl are relatively short lasting and often require people to consume opioids more frequently. If people are using prescription stimulants to control withdrawal symptoms and decrease their frequency of opioid use, this may prove to be a very important finding that merits further exploration.

#### Prescription Stimulant Use Among Unhoused Participants

Others described prescription stimulants as a much-needed tool to help them stay awake, particularly if they were unhoused and living on the street:

Just getting through the day because, you know, being outside, being homeless, like, you know, it’s not like I can just, like, go home and crash on my couch. So, you know, when the next day starts, it’s like, ugh I don't know if I can get through it...taking that Adderall is like the next best thing. Then, coffee I guess [laughs].

[White Hispanic female participant; aged 40 years]

Prescription stimulant use among unhoused individuals warrants particular attention. For safety reasons, people who live on the street need to stay awake. As the participant notes in this quote, she simply does not have the ability to relax at home. Prior research has shown that homeless people frequently use illicit stimulants (eg, methamphetamine) to maintain alertness. If unhoused populations are now also using Ritalin and Adderall, this also warrants further research.

#### Pathways to Initial Use

The people we spoke with reported a variety of paths to using Ritalin or Adderall for the first time, including legitimate prescriptions, prescriptions from physicians whom they paid illicitly, and pills diverted from friends:

It was prescribed while I was in high school...mainly for focus. Just so I could do my high school work and sort of, like, stay focused inside of school and outside of school, because I wouldn't really focus inside of school. And anytime I was outside of school, anything like shiny bright lights, big noises would uh, like, sort of make me lose track of what I'm doing.... But, then after, once I dropped out, I started going into more the illegal side.

[White Hispanic male participant; aged 25 years]

Interviewees described the perceived value and safety of using stimulants sourced directly from a physician or from a trusted person they knew who had a prescription. In this time of adulterated drugs, obtaining legitimate Ritalin or Adderall pills from a reliable source was seen as protection against using adulterated street drugs or even illicit substances such as methamphetamine pressed into pill form and labeled as a prescription stimulant:

I paid a doctor in Queens. I was getting other medication along with the Adderall. It was other medications in the bag, like Percocets and stuff like that.... That’s how I really knew what they were.... So when I couldn't get them from my doctor anymore, I knew to come over here and get them on the black market.

[Black non-Hispanic male participant; aged 49 years]

I got it from friends. Friends that had prescriptions. Just buying it from friends, really, you know, or whoever had it at the time.

[White Hispanic female participant; aged 40 years]

While, for some, initiation to stimulant use was for medical purposes and prescribed by a physician, almost all the participants we spoke with reported ultimately obtaining Ritalin or Adderall illicitly.

## Discussion

### Principal Findings

These findings challenge long-standing social distinctions between “prescription” and “street” stimulants and raise additional questions about how race and class shape access and use. Our research suggests that Ritalin or Adderall use may be increasingly common among lower-income, non-White people in New York City, including individuals who report being homeless. Interviews also show that, instead of use as an off-label study aid or productivity tool for privileged adolescents and emerging adults or treatment for ADHD as directed by a physician, prescription stimulants are also being used to mitigate the effects of fentanyl, xylazine, medetomidine, and other synthetic adulterants in the unregulated drug supply. Data also indicate that people are using Ritalin or Adderall to manage opioid withdrawal and even to limit or curtail their opioid use. These are novel findings that underscore the need to reframe who is using these drugs and why and suggest new possibilities for messaging and outreach.

Our study findings can be interpreted within an adaptive harm reduction framework that views people who use drugs as active agents who continually adapt their behavior in response to changing drug environments rather than as passive recipients of risk [23]. This framework builds on the Risk Environment Framework, where drug-related harms emerge through the interaction of individuals with evolving social, structural, and drug market conditions rather than from individual behavior alone [24,25]. As has been documented with recent shifts from injection to inhalation of opioids, the increasing potency and unpredictability of today’s opioid supply, which includes fentanyl, xylazine, medetomidine, and other adulterants, may suggest that participants are developing a new adaptive strategy: using prescription stimulants to counter oversedation, extend periods between opioid use, or reduce overall opioid use, maintain daily functioning, and manage withdrawal, all of which are participant-generated responses to a rapidly changing drug supply [2,23]. Viewed through this lens, prescription stimulant use is not simply another form of polysubstance use but a potentially adaptive response to an evolving overdose risk environment that warrants further investigation.

As detailed in the Results section, participants who reported concurrently using Ritalin or Adderall and opioids during the previous 30 days were 3.5 times more likely to agree with the statement “I want to stop using drugs but I need help to do that.” This highlights how participants appear to be using stimulants as an adaptive strategy to mitigate drug supply harms and underscores the value of low-threshold outreach delivered with respect and compassion. It also may provide an opportunity to connect people with safer use services and treatment. Our team’s prior research for this project shows that people who use drugs are often reluctant to engage with available safer use services because they fear being treated disrespectfully [6,26] and frequently avoid treatment due to an incorrect belief that they will need to go to a clinic every day for the rest of their lives to receive medication [27]. Further research is warranted to examine how new forms of outreach and intervention content can encourage people who use drugs to connect with compassionate services that are offered in respectful ways they will accept. This includes services offered by trusted community-based organizations and messaging delivered by credible individuals who draw on their own lived experience to discuss the importance of protection against overdose, which includes access to medications for opioid use disorder and other forms of treatment.

Our experimental approach to automated transcription and coding appears promising and offers opportunities for other researchers. Newly available AI tools enabled our research staff to efficiently create and process a set of transcripts, leaving additional time for discussion and thoughtful deliberation among our team as we wrote this manuscript. This does not by any means indicate that important tasks were simply offloaded to AI or that the role of experienced researchers was diminished. We carefully guided the AI and evaluated its output at each step because we believe that successful research requires thoughtful, experienced people working on all phases of a project.

Most importantly, our use of AI allowed us to focus on the strengths of our team members. The 2 researchers who conducted the interviews are especially skilled at approaching underserved people who use drugs and building the trust necessary to address sensitive topics such as illicit substance use. At this time, no existing AI could recruit a comparable sample of participants, establish personal connections, and conduct meaningful face-to-face interviews. Processing our data more efficiently allowed our team to go back into the field more quickly so they could engage in work they are uniquely qualified to do with people who might otherwise not be reached.

### Limitations

The first limitation is that our study recruited a convenience sample of participants and is therefore not intended to be representative of the entire population of people in New York City who use drugs. Similarly, our interviews are not intended to be generalizable beyond our sample. Nonetheless, the fact that participants described the use of prescription stimulants in detail, including how and why they used them and their paths to initial use, suggests new polysubstance use behaviors that remain understudied and deserve further examination.

### Conclusions

Misra et al [18] write that combining traditional qualitative analysis with AI tools offers a “promising innovative strategy” but requires “human oversight to preserve context, nuance, and interpretive depth.” We very strongly agree and, for that reason, emphasize the importance of having a full research team carefully supervise any use of AI.

Just as our goal has always been to help service providers use technology to more effectively work with those in greatest need, our use of new technologies to help facilitate transcription and coding is intended to help researchers more efficiently sort through their data and disseminate key findings. Similarly, conducting additional research to understand why people use prescription stimulants alone and in combination can potentially lead to a more nuanced and inclusive understanding of polysubstance use and what types of messaging or configurations of technology-based intervention content can more effectively encourage people to take steps to protect themselves and others against overdose.

## Supplementary material

10.2196/99947 Multimedia Appendix 1 Coding and analysis prompts for Claude.

10.2196/99947 Multimedia Appendix 2 Project codebook.

## Abbreviations

ADHD: attention-deficit/hyperactivity disorder
PI: principal investigator

## References

[1] Tanz LJ, Miller KD, Dinwiddie AT (2025). Drug overdose deaths involving stimulants - United States, January 2018-June 2024. MMWR Morb Mortal Wkly Rep.

[2] Ciccarone D (2021). The rise of illicit fentanyls, stimulants and the fourth wave of the opioid overdose crisis. Curr Opin Psychiatry.

[3] (2024). Stimulant use and stimulant use disorder in New York State. Office of Addiction Services and Supports.

[4] (2025). Unintentional drug poisoning (overdose) deaths in New York City in 2024. https://www.nyc.gov/assets/doh/downloads/pdf/epi/databrief150-unintentional-drug-death-2025.pdf.

[5] Paradise RK, Desmarais J, O’Malley SE (2023). Perspectives and recommendations of opioid overdose survivors experiencing unsheltered homelessness on housing, overdose, and substance use treatment in Boston, MA. Int J Drug Policy.

[6] Aronson ID, Cramer A, Quiles R, Bennett AS Unpackaging why people who use drugs in New York City decline to connect with harm reduction services or obtain related resources (oral presentation).

[7] Morton WA, Stockton GG (2000). Methylphenidate abuse and psychiatric side effects. Prim Care Companion J Clin Psychiatry.

[8] Danielson ML, Claussen AH, Bitsko RH (2024). ADHD prevalence among U.S. children and adolescents in 2022: diagnosis, severity, co-occurring disorders, and treatment. J Clin Child Adolesc Psychol.

[9] Arria AM, DuPont RL (2010). Nonmedical prescription stimulant use among college students: why we need to do something and what we need to do. J Addict Dis.

[10] Danielson ML, Bohm MK, Newsome K (2023). Trends in stimulant prescription fills among commercially insured children and adults - United States, 2016-2021. MMWR Morb Mortal Wkly Rep.

[11] Morgan PL, Hu EH (2023). Sociodemographic disparities in ADHD diagnosis and treatment among U.S. elementary school children. Psychiatry Res.

[12] Coker TR, Elliott MN, Toomey SL (2016). Racial and ethnic disparities in ADHD diagnosis and treatment. Pediatrics.

[13] Wong SH, Stevens C, Liu CH, Chen JA (2022). Prevalence and correlates of prescription stimulant misuse among US college students: results from a national survey. J Clin Psychiatry.

[14] Board AR, Guy G, Jones CM, Hoots B (2020). Trends in stimulant dispensing by age, sex, state of residence, and prescriber specialty - United States, 2014-2019. Drug Alcohol Depend.

[15] LaBossier NJ, Hadland SE (2022). Stimulant misuse among youth. Curr Probl Pediatr Adolesc Health Care.

[16] Bijker R, Merkouris SS, Dowling NA, Rodda SN (2024). ChatGPT for automated qualitative research: content analysis. J Med Internet Res.

[17] Yue Y, Liu D, Lv Y, Hao J, Cui P (2025). A practical guide and assessment on using ChatGPT to conduct grounded theory: tutorial. J Med Internet Res.

[18] Misra R, Dahal R, Kirk B (2026). Large language models in qualitative analysis: comparing traditional and researcher-interpreted approaches. Int J Qual Methods.

[19] (2010). The ASSIST project - Alcohol, Smoking and Substance Involvement Screening Test. World Health Organization.

[20] NIDA-modified Alcohol, Smoking, and Substance Involvement Screening Test. National Institutes of Health National Institute on Drug Abuse.

[21] Qiao S, Fang X, Wang J, Zhang R, Li X, Kang Y (2025). Generative AI for thematic analysis in a maternal health study: coding semistructured interviews using large language models. Appl Psychol Health Well Being.

[22] Tai RH, Bentley LR, Xia X (2024). An examination of the use of large language models to aid analysis of textual data. Int J Qual Methods.

[23] Bennett AS, McCollum DR, Elliott L (2025). Navigating the COVID-19 risk environment, overdose prevention, and self care practices of people who use illicit opioids in New York City. Subst Use Misuse.

[24] Rhodes T (2002). The ‘risk environment’: a framework for understanding and reducing drug-related harm. Int J Drug Policy.

[25] Rhodes T (2009). Risk environments and drug harms: a social science for harm reduction approach. Int J Drug Policy.

[26] Aronson ID, Bennett AS, Marcus O, Cramer AD, Quiles R, Fong C (2026). Proceedings of the 59th Hawaii International Conference on System Sciences.

[27] Aronson ID, Cramer A, Quiles R, Fong C, Bennett AS New measures designed to assess relationships between substance use related attitudes, knowledge, and engagement with MAT [Poster].

[28] Together Against Overdose: community developed technology to encourage drug checking, distribution of harm reduction supplies, and linkage to services. HEAL Data Platform.

