# Supplementary material for: Examining Increased Ritalin and Adderall Use Among Low-Income People Who Use Drugs: Mixed Methods Study
Source: JMIR Form Res. 2026 Sep 1;10:e99947. doi: 10.2196/99947 (PMC13532686; doi:10.2196/99947)
Supplement: Supplementary file 1 [file formative-v10-e99947-s001.docx]

**Prompt 1:**

You are assisting with qualitative data coding using my CodeBook (draftCodeBook01.docx). Task: Apply the codes from the codebook to the provided transcript.

Rules:

- Only use codes that are explicitly defined in the codebook.
- Do not invent new codes.
- Output must be DOCX.

**Prompt 2:**

the code "Source (e.g. prescription, friend, party)" is a subcode of Context of initial use should refer only to initial use. Any discussion of current supply should be coded under "Sources of current supply."

**Prompt 3**

"Perceived benefits of prescription or other source of supply?" should be applied if the interviewee says they think having a prescription is better, or if they discuss why their source of Ritalin/Adderall is better than other sources

**Prompt 4**

please code additional uploaded transcripts. create new output Word files for each.

**Prompt 5**

based on the codes applied to ALL transcripts, identify a set of themes (codes that appeared in multiple transcripts). create a list of themes, organized by frequency. List the these with the most coded quotes first.

**Prompt 6**

examine each of the excerpts from all the quoted transcripts. organize them into themes based on the content of the excerpts.
