# Supplementary material for: Examining Increased Ritalin and Adderall Use Among Low-Income People Who Use Drugs: Mixed Methods Study
Source: JMIR Form Res. 2026 Sep 1;10:e99947. doi: 10.2196/99947 (PMC13532686; doi:10.2196/99947)
Supplement: Supplementary file 2 [file formative-v10-e99947-s002.docx]

Age at first use

Context of initial use

Reason or desired outcome

Source (e.g. prescription, friend, party)

Was initial use before or after use of other drugs?

Frequency of initial use

Desired outcome(s) of current use

Focus

To balance use of opioids

Changes in desired outcome over time

Other drugs used now

Is Ritalin/Adderall used in combination with other drugs or at different times?

Sources of current supply

Friend

Doctor

Dealer

Perceptions of increased use in community over time?

Reasons for popularity

Addition or dependance

Withdrawal/discomfort

Use of Ritalin/Adderall compared to other stimulants

Perceived benefits of prescription or other source of supply?

Medical insurance

Housing situation

Unhoused

Stably housed

Living in shelter
